# American Visceral Leishmaniasis: Factors Associated with Lethality in the State of São Paulo, Brazil

**DOI:** 10.1155/2012/281572

**Published:** 2012-09-13

**Authors:** Geraldine Madalosso, Carlos Magno Fortaleza, Ana Freitas Ribeiro, Lisete Lage Cruz, Péricles Alves Nogueira, José Angelo Lauletta Lindoso

**Affiliations:** ^1^Centro de Vigilância Epidemiológica “Prof. Alexandre Vranjac”, Coordenadoria de Controle de Doenças, SES, 01246-902 São Paulo, SP, Brazil; ^2^Departamento de Doenças Tropicais e Diagnóstico Por Imagem, Universidade Estadual Paulista Júlio de Mesquita Filho, 18618-970 Botucatu, SP, Brazil; ^3^Departamento de Epidemiologia, Faculdade de Saúde Pública Universidade de São Paulo, 01246-904 São Paulo, SP, Brazil; ^4^Instituto de Infectologia Emílio Ribas, SES, 01246-900 São Paulo, SP, Brazil; ^5^Laboratório de Soroepidemiologia (LIM 38 HC-FMUSP), Instituto de Medicina Tropical Universidade de São Paulo, 05403-000 São Paulo, SP, Brazil

## Abstract

*Objectives*. To identify factors associated with death in visceral leishmaniasis (VL) cases. *Patients and Methodology*. We evaluated prognostic factors for death from VL in São Paulo state, Brazil, from 1999 to 2005. A prognostic study nested in a clinical cohort was carried out by data analysis of 376 medical files. A comparison between VL fatal cases and survivors was performed for clinical, laboratory, and biological features. Association between variables and death was assessed by univariate analysis, and the multiple logistic regression model was used to determine adjusted odds ratio for death, controlling confounding factors. *Results*. Data analysis identified 53 fatal cases out of 376 patients, between 1999 and 2005 in São Paulo state. Lethality was 14.1% (53/376), being higher in patients older than fifty years. The main causes of death were sepsis, bleeding, liver failure, and cardiotoxicity due to treatment. Variables significantly associated with death were severe anemia, bleeding, heart failure, jaundice, diarrhea, fever for more than sixty days, age older than fifty years, and antibiotic use. *Conclusion*. Educational health measures are needed for the general population and continuing education programs for health professionals working in the affected areas with the purpose of identifying and treating early cases, thus preventing the disease evolution towards death.

## 1. Introduction

Visceral leishmaniasis (VL) is a disease with broad geographic distribution, being reported mainly in Asia, Europe, Africa, and the Americas, and is one of the six so-called worldwide priorities among endemic diseases. Five countries report 90% of diagnosed cases in the world [1]. In Brazil, VL is present in five regions, in 21 states. Official data from the Ministry of Health reports approximately two to three thousand new cases per year, and the coefficient of incidence has reached 2 : 100.000 inhabitants. The average fatality rate in the period between 1980 and 2005 was 6.1, peaking at 7.5 in 2004 [2–4]. Some factors contributed to expansion of VL and increase in lethality. Factors related to changes in geographic occurrence pattern result from the intense migration of the rural population to the outskirts of medium and large cities [1]. In addition, the network organization process, associated with better assistance for diagnosis and treatment, resulted in increased detection of cases. Increase of lethality due to VL has been associated with the introduction of the disease in new geographic areas and host factors, such as malnutrition; immunosuppression, mainly HIV coinfection and age extremes [5, 6]. Classically, VL is clinically characterized by hepatosplenomegaly, associated with fever and pancytopenia. The disease is fatal if not treated promptly after the initial symptoms. The most important cause of death in VL patients is, severe anemia, bacterial infections, acute bleeding, sepsis, heart failure, liver failure, and complications arising from the toxicity of antimonials, such as cardiac arrhythmias [6–13].

**Figure 1:**
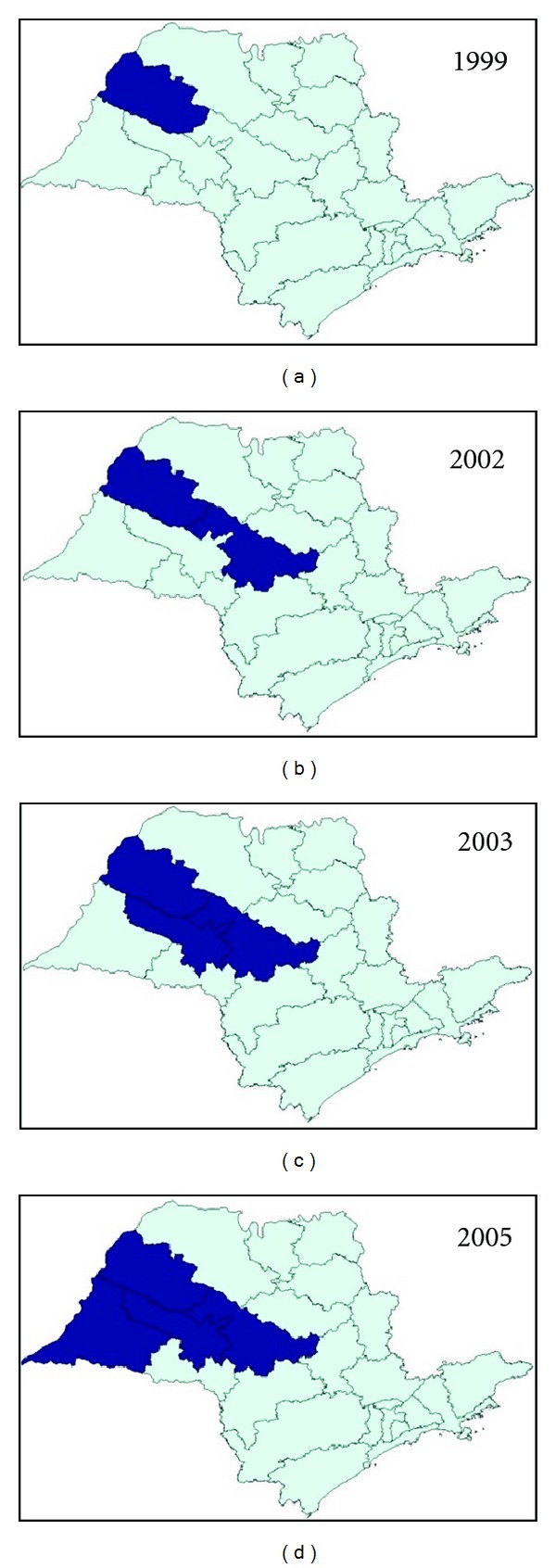
Geographic expansion of confirmed cases of visceral leishmaniasis autochthonous transmission, according to the region of Sao Paulo state, from 1999 to 2005. Data source: SINANW-National Surveillance System for Disease Notification/CVE/SES/SP; TABWIN 3.1.

In the state of São Paulo, until 1998, there were only imported cases of VL from endemic regions. From that year onward, an outbreak of canine visceral leishmaniasis was detected in the western region of the state, and, subsequently, the first human case of VL was diagnosed in 1999. We observed an expansion of VL cases since then: in 2002, the disease was identified in the municipalities located in the region of Bauru and, in 2003, in the region of Marília [14], reaching, until October 2005, 31 municipalities in the regions of Araçatuba, Bauru, Marília, and Presidente Prudente (Figure 1). The VL Control Program from the Brazilian Ministry of Health aims to reduce the rates of morbidity and lethality in humans, by means of diagnosis and early treatment of cases and to decrease the risks of disease transmission through population control of reservoirs and transmitter agents [3, 15]. In order to implement public policies and strategies to improve the clinical management of the disease, as well as epidemiological surveillance programs for the early detection and reduction of lethality, it is necessary to understand the factors associated with the risk of death by AVL. The present study aims to characterize the AVL cases from São Paulo state in the period of 1999 to 2005 and identify factors associated with death.

## 2. Patients and Methods

### 2.1. Study Design

A prognostic study nested in a clinical cohort was carried out by data analysis of 376 medical files. All subjects included in this study were natives from São Paulo state in the period between 1999 and 2005. For analysis of the data, the compulsory disease notification system (SINAN database) was used, which defines the autochthonous cases of visceral leishmaniasis in São Paulo state.

After survey of autochthonous cases and their places of notification, the regional directories of health (DIRs) were asked to identify the unit of hospitalization of the patients. Thus, a specific questionnaire was applied to collect the information from the patients' records. The variables included in the questionnaire were related to demographic, clinical, and laboratory data. After authorization, the hospitals were visited in order to get the patient's records and filling out questionnaires.

### 2.2. Case Definition

A case of human visceral leishmaniasis was defined by clinical and laboratory diagnostic of VL, using direct parasitological examination or culture of specimens collected by venipuncture or biopsy from bone marrow in the routine assessment.

As inclusion criterion, the confirmed cases of AVL had to have São Paulo state as the probable local of infection between 1999 and 2005. 

### 2.3. Variables Analyzed

To identify the probable factors related to lethality, the following variables were used.

1. Biological and demographic variables: age, sex, ethnicity, housing zone, and municipality of residence.
2. The probable site of infection: municipality with proof of transmission of VL in São Paulo state, up to the period in which the study was performed.
3. Care and hospitalization: type of health unit service and date of service.
4. History of current disease: date of symptom onset, presence of the following signs and symptoms: fever, weight loss, abdominal growth, asthenia, headache, abdominal pain, anorexia, constipation, nausea, vomiting, dry cough, dyspnea, drowsiness, myalgia, hemorrhagic manifestations, skin pallor, jaundice, splenomegaly, hepatomegaly, dehydration, adenomegaly, respiratory alterations, and cardio-circulatory changes.
5. Early pathological history or associated conditions: diabetes mellitus with or without organ damage, congestive heart failure, chronic obstructive pulmonary disease, peripheral vascular disease, moderate or severe kidney disease, moderate or severe liver disease, malignant neoplasm, leukemia, lymphoma, and solid metastatic tumor, aids, tuberculosis, malnutrition, immunosuppressive medication, and previous splenectomy.
6. Laboratory tests: levels of hemoglobin, hematocrit, leukocytes, neutrophils, and platelets.
7. Relapse: symptom recurrence up to 12 months after cure, time of relapse (from the end of previous treatment).
8. Patient: clinical evolution of cure or death and cause of death.

### 2.4. Data Collection and Management

An analysis of existing data in the database was conducted based on the variables found in the individual records of epidemiological investigation for human visceral leishmaniasis. A database was developed and analyzed using specific software, Epi-info version 3.2.2, based on questionnaires filled out in consultation together with patient records.

According to the clinical evolution or the type of outcome of confirmed cases of VL, two distinct groups were considered: (1) those who were cured and (2) those who died, in order to identify factors associated with lethality.

### 2.5. Statistical Analysis

The statistical analysis of the data was performed using the software program Epi-info version 3.2.2. Data tabulation was performed using Microsoft Office Excel 2003. After performing the descriptive analysis and determining the main independent variable frequencies, a bivariate analysis was developed between potential risk factors and death outcome. For the continuous quantitative variables, a mean comparison test was performed among the factors assessed through evolution to death or cure. Thus, variables with a *P* value <0.05 were considered for the multivariate logistic regression model by stepwise forward, from the smallest to the largest value of *P*. The existence of an association between death by VL and the factors of interest was investigated by nonadjusted and adjusted odds ratio, with the respective 95% confidence intervals using logistic regression. The statistical significance of variables in the models was assessed by the likelihood ratio test.

## 3. Results

### 3.1. Analysis of Data from SINAN Database, São Paulo State in the Period of 1999 to 2005

During the study period, 945 confirmed cases of VL were reported to the SINAN database, of which 559 were considered autochthonous, with 68 deaths. Lethality reached 14.7% in 2003, with rates remaining at around 11% in the following years.

### 3.2. Analysis of Data Collected from Patient Records

In order to determine the prognostic factors of VL and identify the risk of lethality 376 patient records of 559 autochthonous cases of VL that had an outcome (67.3%) were assessed. Two separate groups were studied and analyzed according to the patient's clinical evolution: the cases that resulted in death and cases in which the disease was cured. Of 376 cases assessed, 53 resulted in death and 323 in cure. Lethality observed in this group in the study period was 14.1%. The distribution by age groups reflects the predominance of cases among children younger than 10 years (53.7%), while the greater lethality is in the age groups older than 50 years, particularly in those older than 60 years (69%), as demonstrated in Table 1. There was a predominance of males, especially in the age groups older than 10 years. Regarding the signs and symptoms described in patients' records, fever, splenomegaly, hepatomegaly, pallor, asthenia, and weight loss were the most frequent (≥60), and proportional in both study groups (Table 2). Other less frequent findings, such as dry cough, diarrhea, dehydration, hemorrhagic manifestations, edema, and jaundice, were proportionally more frequent in cases that resulted in death. Malnutrition was seldom described in patients' records (1.6%). The age of patients varied from 3 months to 96 years (average = 19 years), with difference between cases that resulted in death and cured patients (44 × 15 years; *P* < 0.001). The mean time between symptom onset and treatment was 34 days (51 × 21 days; *P* < 0.001). Comorbidities were present in 72 patients (19.1), and the most frequent was HIV coinfection (*n* = 5). As for the laboratory data, we observed a mean level of hemoglobin of 8.2 g/dL, 3,390 leukocytes/mm^3^, 1,319 neutrophils/mm^3^, and 107,000 platelets/mm^3^, demonstrating the characteristic thrombocytopenia observed in these patients. The main causes associated with death were sepsis (21/53), bleeding (12/53), liver failure (9/53), and arrhythmia due to antimonial cardiotoxicity (9/53). The bivariate analysis of clinical findings and laboratory data associated with worse prognosis of VL cases is shown in Table 3. The final model analysis using multivariate logistic regression (Table 4) showed to be more strongly associated with VL lethality regardless of the other variables: cardiac abnormalities on admission or during hospital stay (OR = 4.7), presence of diarrhea (OR = 2.7), presence of severe anemia identified by hemoglobin ≤5.0 g/dL (OR = 4.5), increase in total bilirubin ≥2.0 (OR = 7.3), age ≥ 50 years (OR = 29.5), time between fever onset and treatment >60 days (OR = 6.2), and use of antimicrobials during hospitalization (OR = 5.7). Hemorrhagic manifestations remained in the final model only as adjustment variables for the remaining ones, as there was no statistical significance (OR = 2.6).

## 4. Discussion

Visceral leishmaniasis is a disregarded emerging tropical disease, which shows modifications in its epidemiological behavior, occurring in new areas [5, 10]. The increase in human VL cases in endemic regions, or even the emergence of the disease in regions where it did not previously occur, can be explained by the change in geographical occurrence patterns, with the reporting of cases in urban centers [16–19]. As reported by other authors, we clearly observe that the introduction of VL in São Paulo state caused an initial increase in lethality, likely by the misdiagnosis of the disease by health care professionals, and, subsequently, the maintenance of fatality levels came as a result of the occurrence of the disease in vulnerable populations, such as those infected with HIV. VL lethality in São Paulo state during the study period was 14.1%, whereas it was 6.7% in the rest of Brazil. The data analysis showed that the quality of the data and mainly the lack of update were limiting factors. On the other hand, it was observed that the vast majority of patients examined came from urban areas and had access to health services and, even so, there was a delay in the diagnosis. The high incidence of the disease in the age groups younger than 10 years observed in data analysis of patients' records corresponds to that found in the literature and the official data of the Ministry of Health. The prevalence of males observed in the most predominant age groups is also a constant characteristic found in several studies [16–19]. Regarding age, the study found high levels of lethality in those aged 50 years and older, with the mean age in the group of patients who died of 44 years, while the mean age in the group of patients that were cured was 15 years. The greatest strength of association was found between the evolution to death and age 50 years and older (OR = 20.16; *P* < 0.0001). The association between death and age with a cutoff of 30 years was also statistically significant, but with less strength (OR = 7.25; *P* < 0.001). Symptoms and clinical findings pointed to severity, and, therefore, factors associated with death, such as evolution to hemorrhagic manifestations, edema, and jaundice, were highlighted in the study, as shown by the largest percentage in the group of deaths [7–13]. 

The classic symptoms, such as fever, hepatosplenomegaly, and pallor, showed no statistical difference between the two groups, as they are not criteria of diagnostic suspicion that influence disease prognosis, and therefore should be equally present in the two groups [9, 11–13]. Other signs and symptoms associated with death, such as cardiac alterations at admission or during hospitalization, dehydration, diarrhea, vomiting, abdominal pain, dyspnea, dry cough, and drowsiness, are also already described in study of risk factors for death. Concomitant diseases and conditions associated with the diagnosis of VL that contributed to the death outcome were moderate liver disease and severe cardiovascular diseases, tuberculosis, and the use of immunosuppressive drugs; these conditions are already known to be risk factors, involving mainly immunity impairment [9–13]. HIV coinfection increased the risk of death but did not show statistical significance, probably by the small number of cases and fatalities evaluated. It is known that this coinfection is emerging and is considered to be of high severity [10, 13, 20]. 

The major complications of the disease found in the study, associated with worse prognosis, corroborate those already described in the literature: bacterial infections, such as pneumonia, skin infections and ear infections, bleeding, and sepsis [8–13, 21]. The use of antimicrobials and the need for blood transfusions and blood products were also associated with greater lethality alone, reflecting the severity of cases and, consequently, the development of complications. Febrile neutropenia and adverse reactions to antimonials did not show statistical significance for the death outcome, although it has been discussed in other studies as factors of poor prognosis. Still, one of the leading causes of death was found to be cardiac arrhythmias due to antimonial use toxicity. Sepsis, bleeding, and liver failure were also causes of death, compatible with the data from the literature [9, 10]. In addition to these, medullary aplasia and aids appeared as causes of death among the evaluated patients.

Laboratory alterations that indicated disease severity, compatible with the literature, were also associated with deaths analyzed in the study. Thrombocytopenia (≤100,000 mm^3^), severe anemia, hypoalbuminemia, hyperbilirubinemia, in addition to the increase in aspartate aminotransferase (AST) levels were associated with statistical significance for the outcome of death. Leukopenia and neutropenia showed unexpected statistically significant differences, requiring further analysis when relating them to other events, where opportunistic infections constitute a complication and not lethality cause [10, 12, 13]. 

Factors associated with the risk of death in the multivariate analysis were the presence of diarrhea and hemorrhagic phenomena, cardiac abnormalities on admission or during hospitalization and treatment, hemoglobin levels ≤5.0 g/dL, and total bilirubin ≥2.0 g/dL, constituting serious anemia and jaundice, respectively. All of these factors have already been addressed in studies as associated with worse prognosis of the disease. Still, the need for the use of antimicrobials as predictor of death, notably in the multivariate analysis, reflects the concomitant infections and results from the severity of the disease; these variables did not remain in the final model, as they represent the same nature of the outcome in question, that is, death. In other words, if antibiotics are used, the reason is the presence of infection, or pneumonia, or otitis, or sepsis, and, conversely, if there is infection, then the use of antimicrobials is required.

Long-term symptoms were found to be associated with greater lethality, a factor that reflects the delay in the detection of cases and areas of recent VL diagnosis in addition to the lack of knowledge on the part of the population that inhabits these periurban and urban areas, as they have universal access to health services through the public health system. The conclusions to be drawn from this study are aimed at the implementation of intervention measures, prevention, and control in certain regions of the state, for certain sectors of the population, with specific clinical findings of paramount importance for the reduction of lethality due to visceral leishmaniasis in the state of São Paulo in Brazil.

## 5. Conclusions

The appearance of visceral leishmaniasis in the state of São Paulo is recent, dating from the year 1999, being considered an emerging disease in our state.

High incidence of cases was observed in children under 10 years and high lethality in those older than 50 years. 

Average lethality for the state of São Paulo in the period 1999 to 2005 was 14.1, which is considered high in comparison with the epidemiological data from other states and even in Brazil as a whole, which shows up to 7.0 of lethality.

Deaths must be investigated to improve the clinical management of cases, through the knowledge of their determining factors and population groups at greatest risk, in addition to increase the epidemiological surveillance system.

The prognostic factors that were more strongly associated with death, regardless of all other variables were major anemia, hemoglobin ≤5.0 g/dL, hemorrhagic manifestations, cardiac abnormalities at admission or during hospitalization and treatment, total bilirubin level <2.0 g/dL, diarrhea, age >50 years or older, time period between symptom onset, indicated by the onset of fever, and treatment longer than 60 days and the need of antimicrobials.

Investment in health education of the population and continuing education programs for health professionals working in the affected areas is of paramount importance for the early detection of cases, thus preventing the evolution towards death.

## Figures and Tables

**Table 1:** Visceral leishmaniasis-confirmed autochthonous cases, by age group and clinical outcome, state of Sao Paulo, 1999–2005.

Age group (years)	Death		Cured		Total		
	*n*	%	*n*	%	*n*	%	% Cumulative
<1	3	7.0	40	93.0	43	11.4	11.4
1–4	8	6.5	115	93.5	123	32.7	44.1
5–9	1	2.8	35	97.2	36	9.6	53.7
10–19	2	6.7	28	93.3	30	8.0	61.7
20–29	3	8.6	32	91.4	35	9.3	71.0
30–39	4	13.8	25	86.2	29	7.7	78.7
40–49	4	11.4	31	88.6	35	9.3	88.0
50–59	8	50.0	8	50.0	16	4.3	92.3
≥60	20	69.0	9	31.0	29	7.7	100.0

Total	53	14.1	323	85.9	376	100	

**Table 2:** Visceral leishmaniasis distribution (*n* = number and %) of the main signs and symptoms, according to the clinical outcome of the confirmed cases, state of Sao Paulo, 1999–2005.

Signs and symptoms	Death (*n* = 53)		Cured (*n* = 323)		Total (*n* = 376)	
	*n*	%	*n*	%	*n*	%
Fever	51	96.2	320	99.1	371	98.7
Splenomegaly	50	94.3	319	98.8	369	98.1
Hepatomegaly	44	83.0	244	75.5	288	76.6
Pallor	39	73.6	199	61.6	238	63.3
Asthenia	44	83.0	191	59.1	235	62.5
Weight loss	35	66.0	189	58.5	224	59.6
Dry cough	29	54.7	127	39.3	156	41.5
Diarrhea	20	37.7	52	16.1	72	19.1
Hemorrhagic manifestations	18	34.0	22	6.8	40	10.6
Edema	10	18.9	11	3.4	21	5.6
Dehydration	12	22.6	8	2.5	20	5.3
Cardiac abnormality	9	17.0	10	3.1	19	5.1
Jaundice	10	18.9	6	1.9	16	4.3
Malnutrition	2	3.8	4	1.2	6	1.6

**Table 3:** Statistical analysis of factors associated with VL lethality, state of Sao Paulo, 1999–2005 (bivariate analysis).

	Outcome (*n* = 376)				
Features	Death	Cured	Total	OR (95% CI)	*P* value
	*n* = 53	*n* = 323	*n* = 376		
Signs and symptoms					
Asthenia	44	191	235	3.38 (1.59–7.16)	0.0008
Cardiac abnormality	9	10	19	6.53 (2.28–18.65)	<0.0001
Dehydration	12	8	20	12.11 (4.66–31.47)	<0.0001
Diarrhea	20	52	72	3.16 (1.68–5.93)	0.0002
Dyspnea	12	22	34	4.00 (1.84–8.69)	0.0001
Edema	10	11	21	6.75 (2.7–16.86)	<0.0001
Hemorrhagic manifestation	18	22	40	7.24 (3.54–14.83)	<0.0001
Jaundice	10	6	16	12.88 (4.45–37.31)	<0.0001
Pallor	39	199	238	2.02 (1.02–4.01)	0.040
Dry Cough	29	127	156	1.86 (1.04–3.35)	0.035
Vomiting	13	35	48	2.67 (1.30–5.48)	0.006
Drowsiness	9	11	20	5.8 (2.27–14.79)	<0.0001

Laboratory analysis					
Total bilirubin ≥ 2.0	13	6	19	17.17 (6.18–47.7)	<0.0001
Hypoalbuminemia ≤ 3.0	15	37	52	3.05 (1.53–6.07)	0.0009
Thrombocytopenia ≤ 100.000	31	108	139	2.8 (1.55–5.08)	0.0004
Aspartate aminotransferase > 40	20	62	82	2.55 (1.37–4.74)	0.002

Comorbidities					
Liver disease	3	2	5	9.79 (1.59–60.11)	0.020
Diabetes	5	4	9	8.46 (2.19–32.62)	0.003
Peripheral vascular disease	7	5	12	9.86 (3.0–32.4)	<0.0001
Splenectomy	2	0	2	undefined	0.019
Congestive heart failure	4	2	6	13.33 (2.38–74.78)	0.004
Use of immunosuppressive drugs	3	0	3	undefined	0.002
Tuberculosis	3	1	4	19.65 (2.0–192.74)	0.009

Fever					
≥60 days	13	26	39	3.71 (1.76–7.80)	0.0002
≥30 days	26	79	105	2.97 (1.64–5.39)	0.0002

Age					
≥30 years	36	73	109	7.25 (3.85–13.66)	<0.0001
≥50 years	28	17	45	20.16 (9.74–41.73)	<0.0001

Complications	53	64	117	undefined	<0.0001
Opportunistic infections	21	43	64	4.27 (2.26–8.08)	<0.0001
Pneumonia	13	29	42	0.40 (0.18–0.89)	0.023
Bleeding	24	2	26	26.1 (5.77–117.78)	<0.0001
Sepsis	28	0	28	undefined	<0.0001
Antimicrobial use	34	59	93	8.71 (4.56–16.64)	<0.0001
Blood derivatives	36	95	131	5.61 (2.93–10.72)	<0.0001

**Table 4:** Final model of factors associated with VL lethality, state of Sao Paulo, 1999–2005 (multivariate analysis).

	Clinical outcome				
Variable	Death	Cured	Total	OR (95% CI)*	*P* value**
	*n* = 53	*n* = 323	*n* = 376		
High total bilirubin					
Total Bilirubin ≥ 2.0 g/dL	13	6	19	7.36 (1.65–32.76)	<0.0001
Severe anemia					
Hemoglobin ≤ 5.0 g/dL	7	13	20	4.56 (1.17–17.48)	<0.0001
Antimicrobial agents	34	59	93	5.76 (2.27–14.64)	<0.0001
Age ≥ 50 years	28	17	45	29.54 (10.6–82.6)	
Length of illness (days)					
Fever >60 days	13	26	39	6.23 (2.05–18.92)	<0.0001
Hemorrhagic manifestations	18	22	40	2.62 (0.93–7.4)	0.0001
Cardiac abnormality	9	10	19	4.73 (1.3–17.23)	<0.0001
Diarrhea	20	52	72	2.76 (1.03–7.43)	<0.0001

*Odds ratio (95% confidence interval). **Likelihood ratio.
